# Supplementary material for: Enhancing calmodulin binding to cardiac ryanodine receptor completely inhibits pressure-overload induced hypertrophic signaling
Source: Commun Biol. 2020 Nov 26;3:714. doi: 10.1038/s42003-020-01443-w (PMC7691336; doi:10.1038/s42003-020-01443-w)
Supplement: Supplementary file 1 — Supplementary Information [file 42003_2020_1443_MOESM1_ESM.pdf]

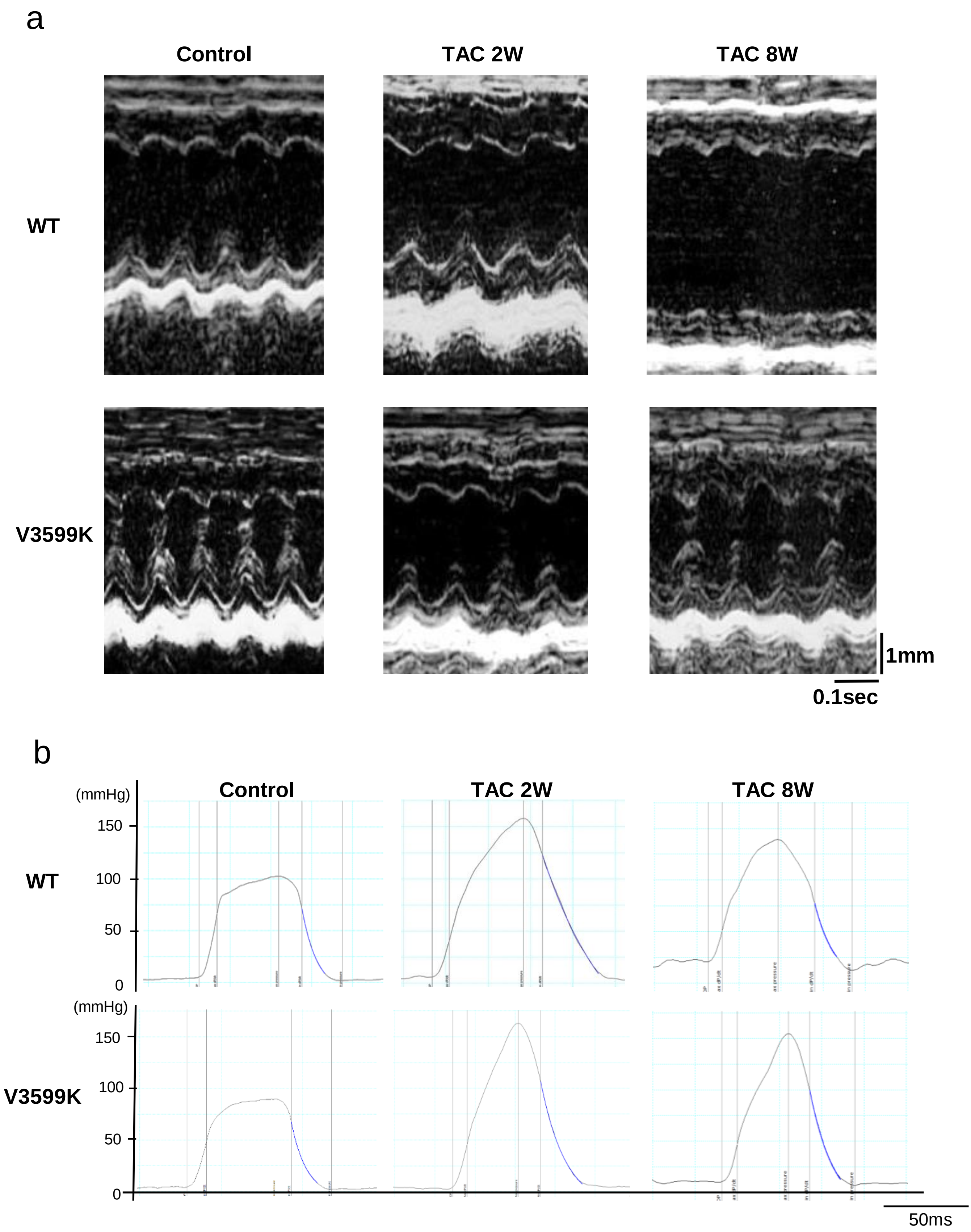

Supplementary Figure 1.
Representative images of cardiac echocardiogram (a) and LV pressure (b).
In WT mice, LV was markedly enlarged with reduced wall motion 8 weeks after TAC, but not in V3599K mice. Although peak LV pressure was similarly increased after TAC in WT and V3599K mice, relaxation time was much more prolonged in WT mice than in V3599K mice.

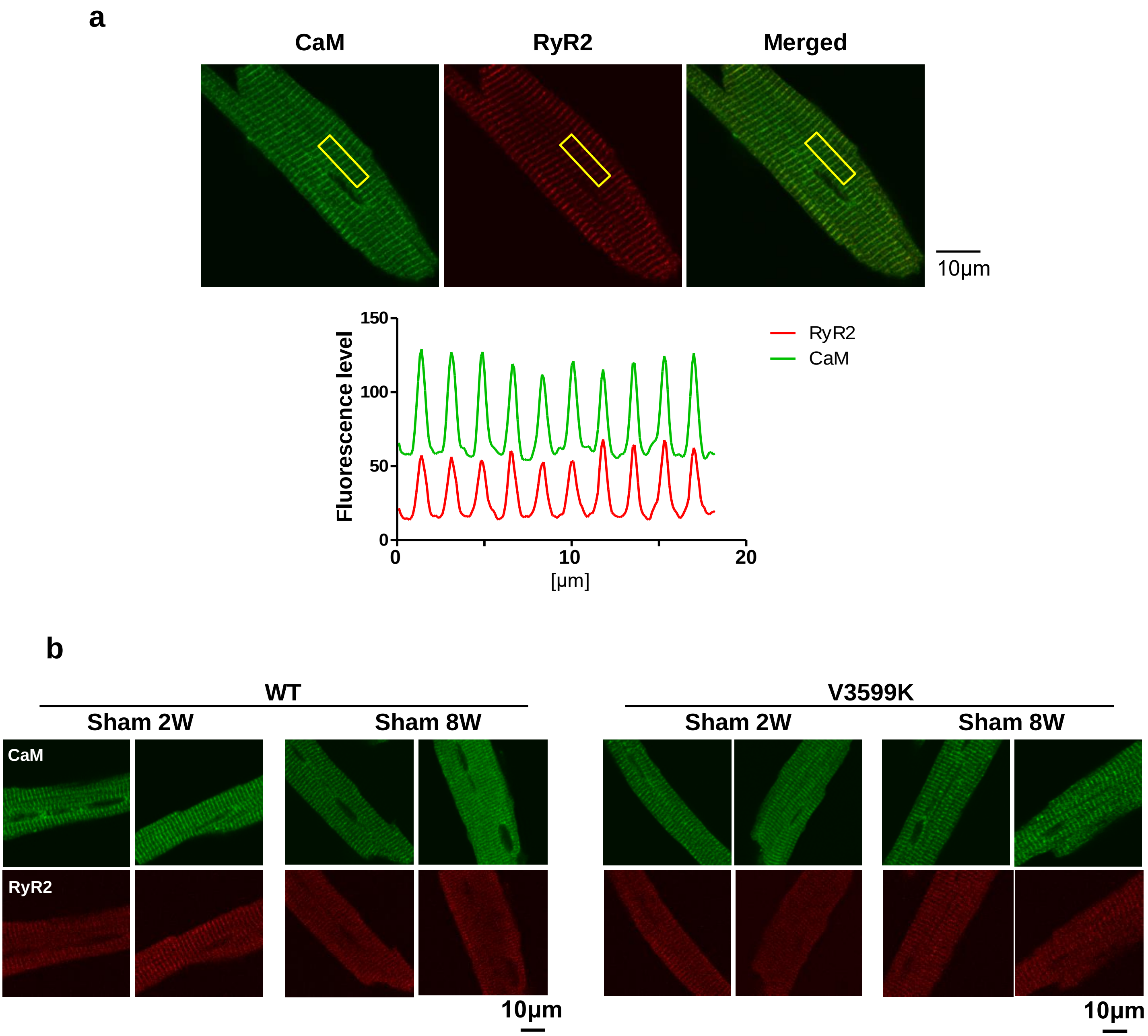

Supplementary Figure 2.
Localization and binding characteristics of endogenous CaM in intact WT and V3599K cardiomyocytes. (a) (Left) Representative images of endogenous CaM, co-localized with RyR2 in WT cardiomyocytes. Immuno-staining of CaM (red); middle; immuno-staining of RyR2 (green). (Right) Merged image. (Right) Periodical increases in Alexa fluorescence (Alexa Fluor 488–conjugated goat anti-rabbit (A11008, Thermo Fisher Scientific, Tokyo, Japan) and an Alexa Fluor 633–conjugated goat anti-mouse (A21052, Thermo Fisher Scientific, Tokyo, Japan) signals of either CaM (red) or RyR (green). CaM was detected along with sarcomeres, showing an excellent co-localization with RyR2. **(b)** Representative images of endogenous CaM co-localized with RyR2 between control (before TAC), Sham 2W, and Sham 8W, as negative controls.

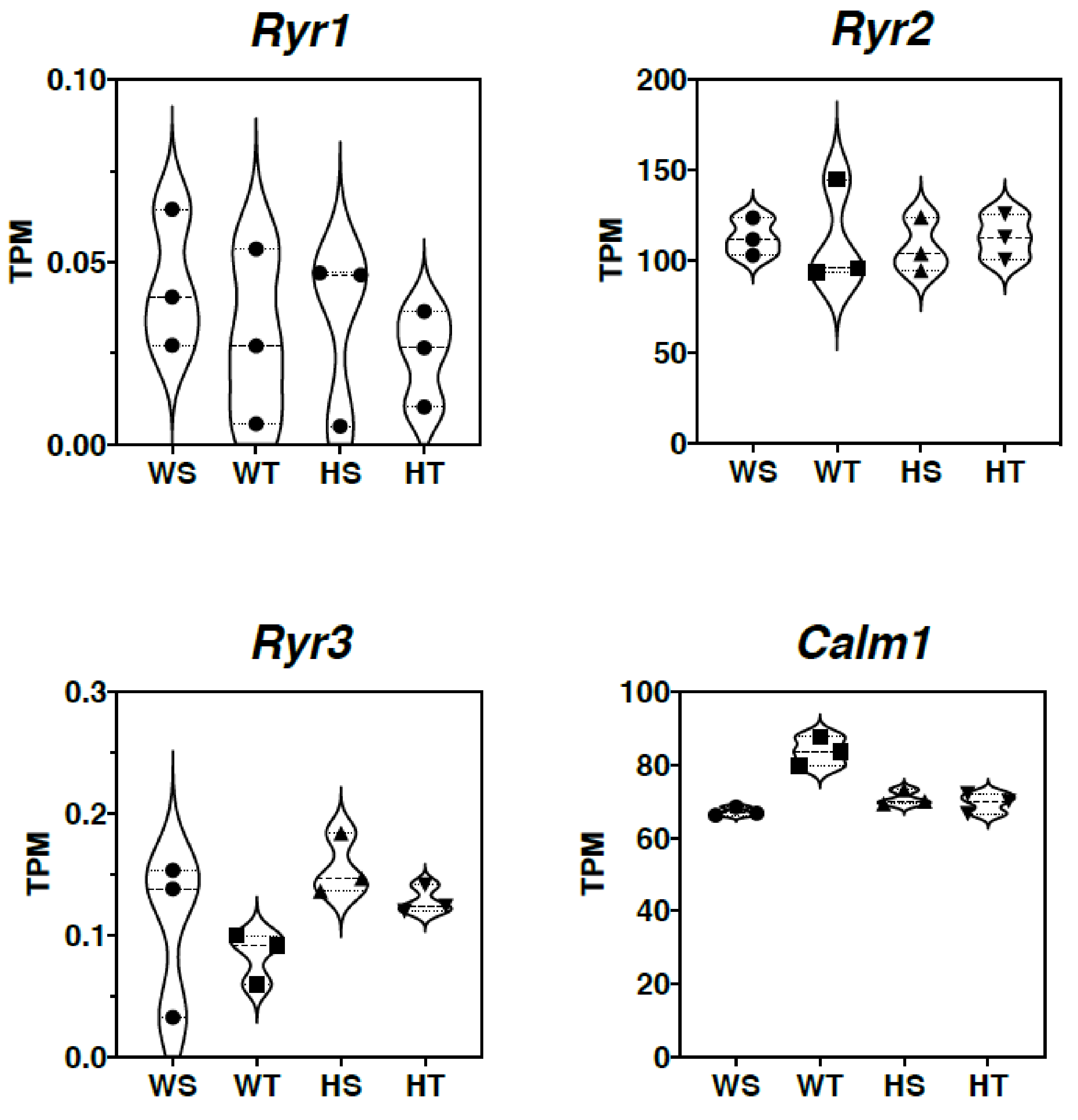

Supplementary Fig.3

Violin plots of gene expression in *RyR* superfamily and *Calm1* in hearts of TAC model mice.

The gene expression in hearts of WT mice without (WS:n=3) or with TAC (WT:n=3) and V3599K mice without (HS:n=3) or with TAC (HT:n=3) was calculated from reads counts of the indicated genes, and was normalized by transcripts per million (TPM).

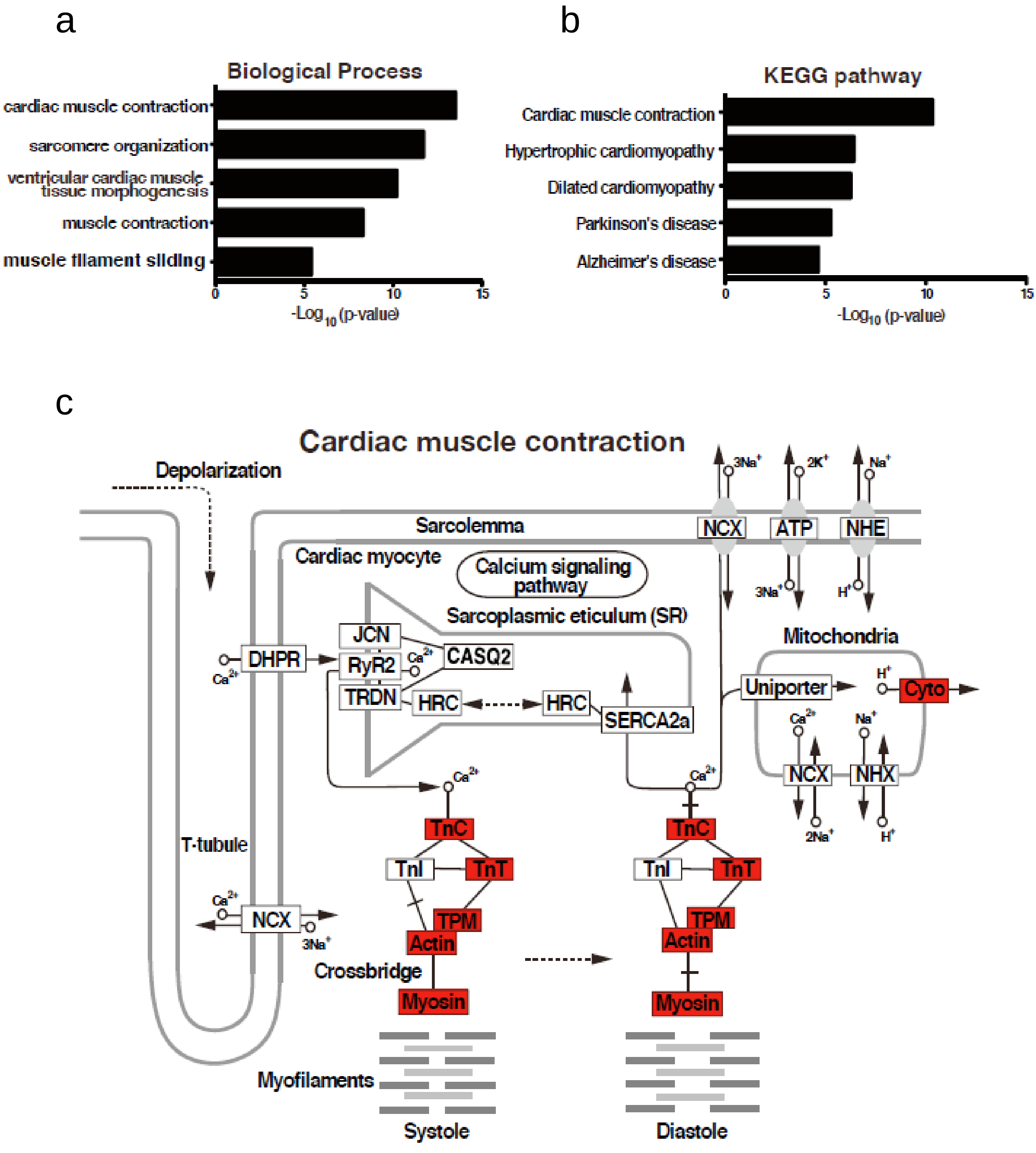

Supplementary Fig.4

Analysis of Gene ontology (GO) and KEGG pathway after chronic pressure-overload of hearts in TAC model mice.

The upper 100 genes of the factor loadings detected in PC3 of PCA using the gene expression in WT mice {without (WS: n = 3) or with TAC (WT: n = 3) } and V3599K mice {without (HS: n=3) or with TAC (HT: n = 3)} were analyzed by GO (a) and KEGG (b) software, and the pathway of cardiac muscle contraction was shown as a top score in KEGG analysis (c).

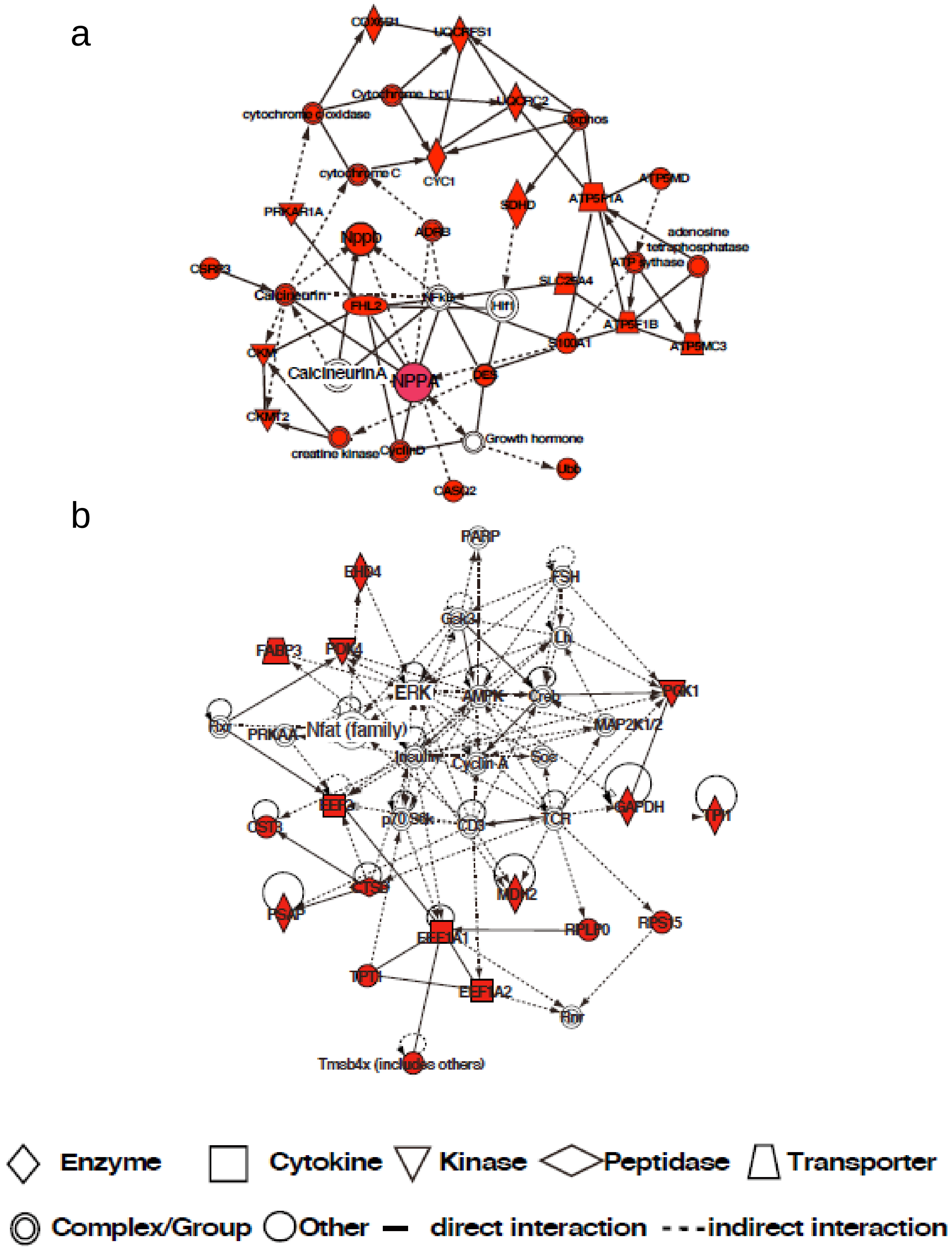

Supplementary Fig.5

Analysis of IPA network pathways after chronic pressure-overload of hearts in TAC model mice.

The upper 100 genes of the factor loadings detected in PC3 of PCA using the gene expression in WT mice {without (WS: n = 3) or with TAC (WT: n = 3) } and V3599K mice {without (HS: n=3) or with TAC (HT: n = 3)} were used for IPA pathway analysis. The network pathways detected as a top score (a) and third score (b) were shown.

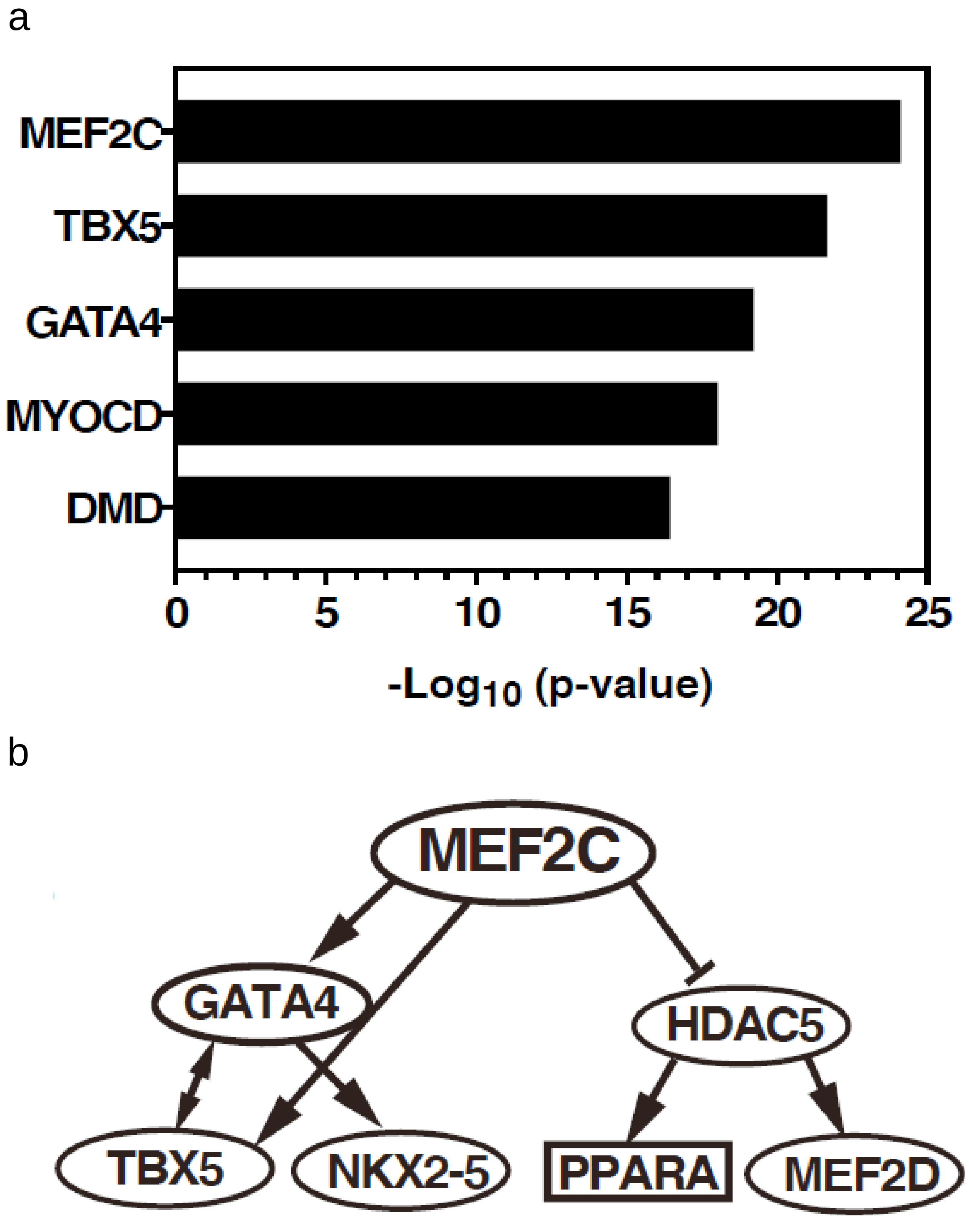

Supplementary Fig.6

Upstream molecules in IPA network analysis after chronic pressure-overload of hearts in TAC model mice.

The upper 100 genes of the factor loadings detected in PC3 of PCA using the gene expression in WT mice {without (WS: n = 3) or with TAC (WT: n = 3) } and V3599K mice {without (HS: n=3) or with TAC (HT: n = 3)} were used for IPA pathway analysis. The upstream molecules were indicated from data of the gene expression (a). The signaling pathway of MEF2C detected as a top score of upstream molecules was shown (b).

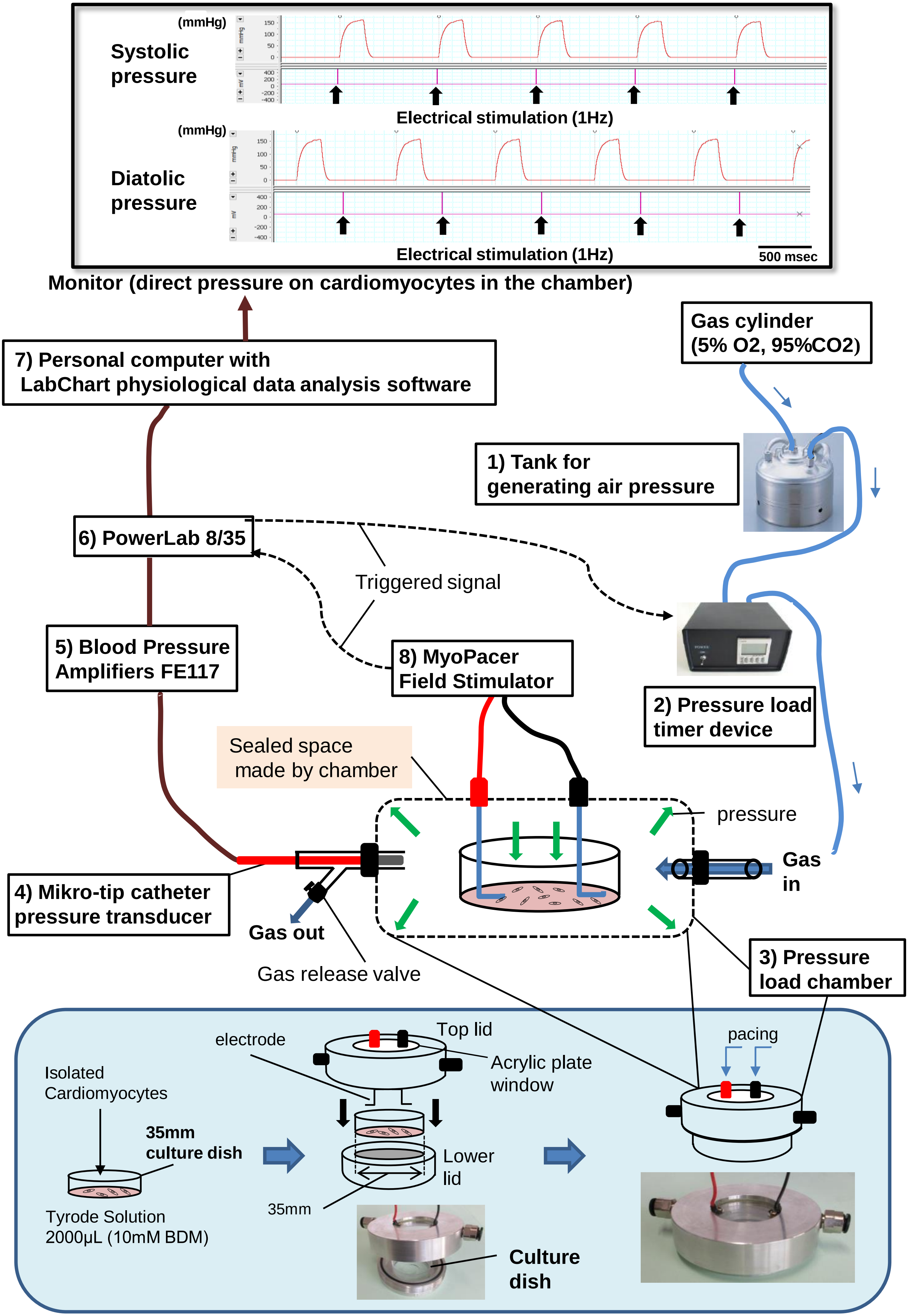
(mmHg)
Systolic pressure
Electrical stimulation (1Hz)
(mmHg)
Diatolic pressure
Electrical stimulation (1Hz)
500 msec
Monitor (direct pressure on cardiomyocytes in the chamber)
Gas cylinder (5% O2, 95%CO2)
7) Personal computer with LabChart physiological data analysis software
1) Tank for generating air pressure
6) PowerLab 8/35
Triggered signal
5) Blood Pressure Amplifiers FE117
8) MyoPacer Field Stimulator
2) Pressure load timer device
Sealed space made by chamber
pressure
Gas in
4) Mikro-tip catheter pressure transducer
Gas out
Gas release valve
3) Pressure load chamber
electrode
Top lid
Acrylic plate window
pacing
Isolated Cardiomyocytes
35mm culture dish
Lower lid
35mm
Tyrode Solution 2000μL (10mM BDM)
Culture dish

Supplementary Figure 7. Acute pressure-overload system by air compression applied to isolated cardiomyocytes.

Monitor shows representative pressure waves due to systolic pressure load (upper) and diastolic pressure load (lower) triggered by electrical stimulation (1Hz).

The mixed gas (5% O_2, 95% CO_2) compression system on cardiomyocytes is composed of **1)** tank made of stainless steel for generating air pressure (SKBT-5L, LABTEC, Fukuoka, Japan) **2)** pressure load timer device (SKPTC-H5CZ, LABTEC, Fukuoka, Japan) **3)** Stainless steel chamber for direct pressure load on cultured cardiomyocytes (SKPTC-35, LABTEC, Fukuoka, Japan) **4)** Mikro-Tip Catherer Pressure Transducers (Millar Instruments, Inc, Houston, USA) **5)** Blood Pressure Amprifiers (FE117 AD Instruments, Colorado Springs, USA) **6)** PowerLab 8/35 (AD Instruments, Colorado Springs, USA) **7)** Personal computer with LabChart physiological data analysis software (AD Instruments, Colorado Springs, USA) **8)** MyoPacer Field Stimulator (ION Optix, Westwood, USA).

The tank connected to the high pressure gas cylinder (5% O_2, 95%CO_2) can freely control magnitude of the pressure load on the cardiomyocytes in the chamber, and the pressure load timer device can freely control the duration and timing of the pressure load by opening or closing the valve in the apparatus, which is triggered by electrical stimulation from MyoPacer Field Stimulator. The controled air pressure is delivered into single entrance of the pressure load chamber.

As shown in the lower panel, 100 to 200 isolated cardiomyocytes attached to the laminin-coated 35mm-diameter glass culture dish are incubated in 2000 μl of Tyrode's solution in the presence 2, 3-butanedione monoxime (BDM) to eliminate the effect of contraction on hypertrophic signaling. The custom pressure load chamber consists of a stainless steel top and bottom lid. The outer diameter of the chamber is 75 mm and the inner diameter is 35 mm. The 35 mm-diameter dish is exactly put in the lower lid and the top lid is firmly fixed. The cardiomyocytes in the glass culture dish are sealed in a stainless steel chamber, so the pressure throughout the cardiomyocytes is kept uniform. There is no cardiomyocyte movement during the acute pressure load on the cardiomyocytes. Mikro-Tip Catherer Pressure Transducers (Millar Instruments, Inc, Houston, USA) is set in the sealed space made of the pressure load chamber. Therefore, the magnitude of the pressure load on the cardiomyocytes in the chamber is accurately measured by an established catheter-tip manometer method, and monitored by a personal computer via Blood Pressure Amplifiers PE117 and PowerLab 8/35. Pressure-overload (150 mmHg, 250 ms) was applied to cardiomyocytes with 1 Hz electrical pacing during the (systolic) Ca^{2+} transient phase or (diastolic) static phase. Ca^{2+} sparks were measured after 10 minutes of pressure overload. The acquisition and analysis of the data were conducted by LabChart (physiological data analysis software).

a

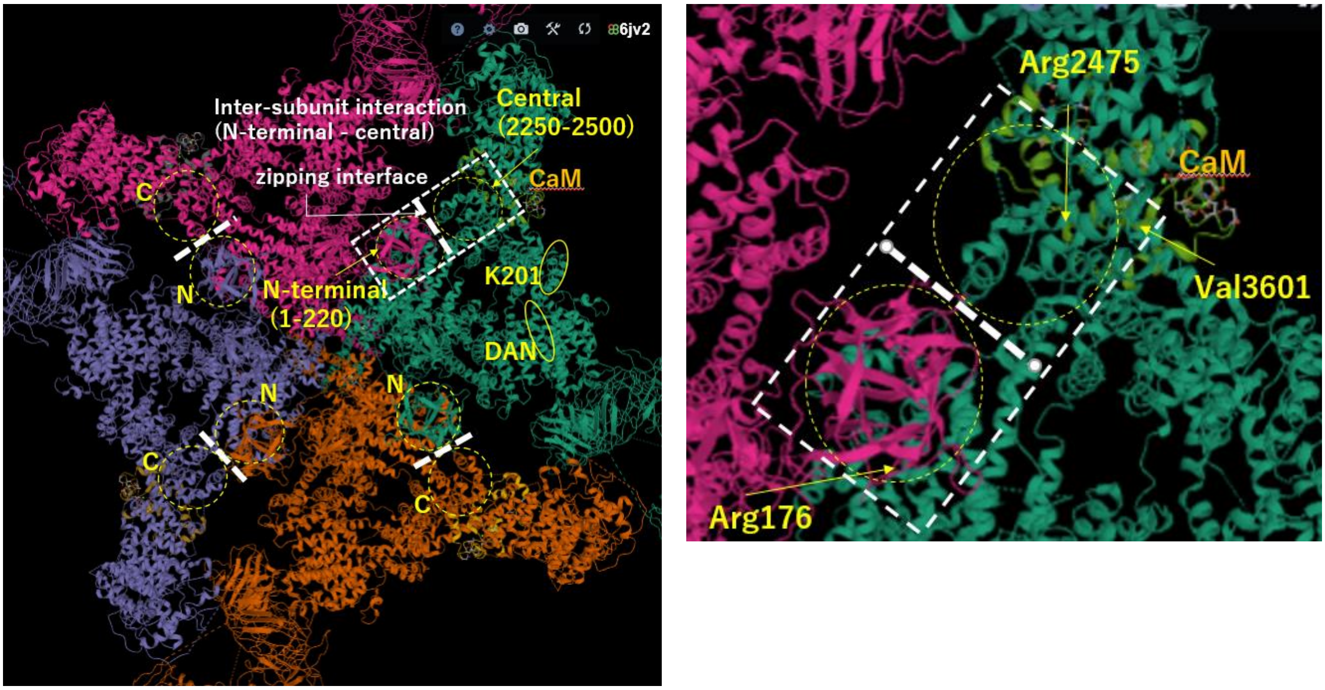

b

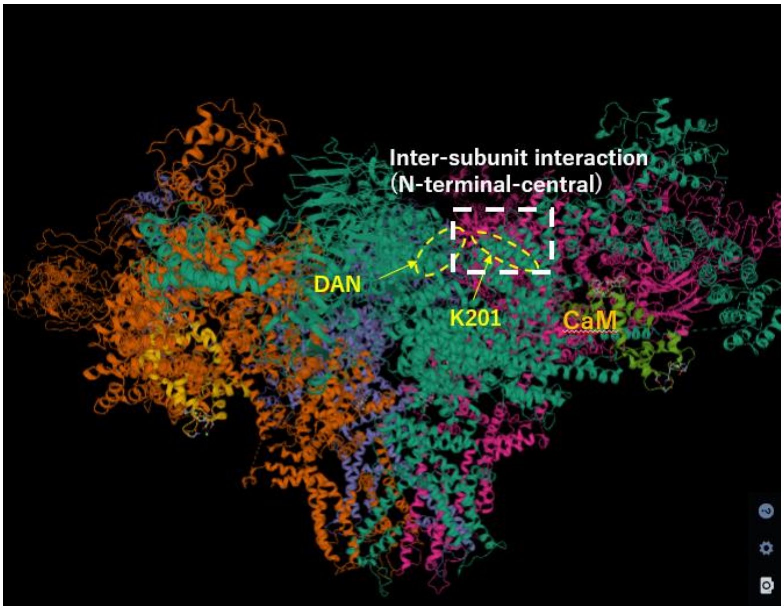

Supplementary Figure 8.
Inter-domain cross-talk between zipping interface and CaM binding site in RyR2 (3D structure was referred from PDB ID: 6JV2, ref 15). Plan view (a) and side view (b) of RyR2 as a tetramer. The interface between N-terminal (1-220: red) and central (2250-2500: green) domains forms an inter-subunit interaction, and closely located to the CaM binding domain (3583–3603), to which CaM (yellow green) binds. Interestingly, the binding site of dantrolene (601-620) (25) is just adjacent to that of another RyR2 stabilizer, K201 (JTV519) (2114-2149) (30).

Supplemental Table1 Upstream analysis of upper 100 gene in factor loadings of PC3 in mice heatrs with TAC

Upstream Regulator	Molecule Type	p-value of overlap	Target molecules in dataset
MEF2C	transcription regulator	7.70E-25	ACTA1,ACTC1,ACTN2,CASQ2,CKM,COL1A1,COL3A1,DES,HSPB7,LMOD2,MYH6,MYH7,MYL2,MYOM1,NPPA,POSTN,TNNC1,TNNT2,VIM
TBX5	transcription regulator	2.22E-22	ACTC1,ACTN2,ANKRD1,CASQ2,COL1A1,COL3A1,DES,HSPB7,MYH6,MYL2,NPPA,POSTN,TNNC1,TNNT2,VIM
GATA4	transcription regulator	6.03E-20	ACTA1,ACTC1,ACTN2,ANKRD1,CASQ2,COL1A1,COL3A1,DES,HSPB7,MYH6,MYH7,MYL2,NPPA,Nppb,POSTN,TNNC1,TNNT2,VIM
MYOCD	transcription regulator	9.55E-19	ACTA1,ACTC1,ACTN2,CASQ2,COL1A1,COL3A1,DES,HSPB7,MYH6,MYH7,MYL2,NPPA,TNNC1,TNNT2
DMD	other	3.68E-17	ACTG1,ANKRD1,BGN,CKMT2,COL3A1,EEF1A1,FTH1,HSPA8,HSPB6,LYZ,MYBPC3,MYH7,PDK4,POSTN,Tmsb4x (includes others),TPI1,VIM

WT Sham 2W

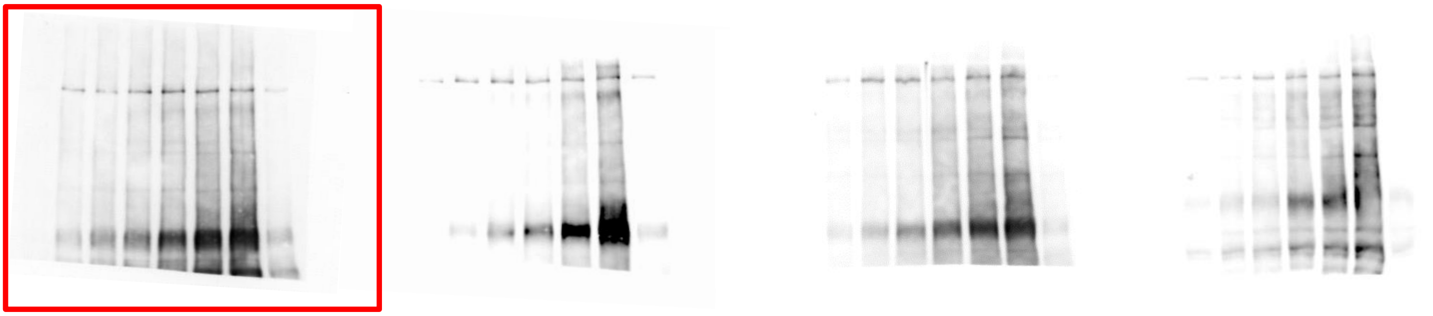

V3599K Sham 2W

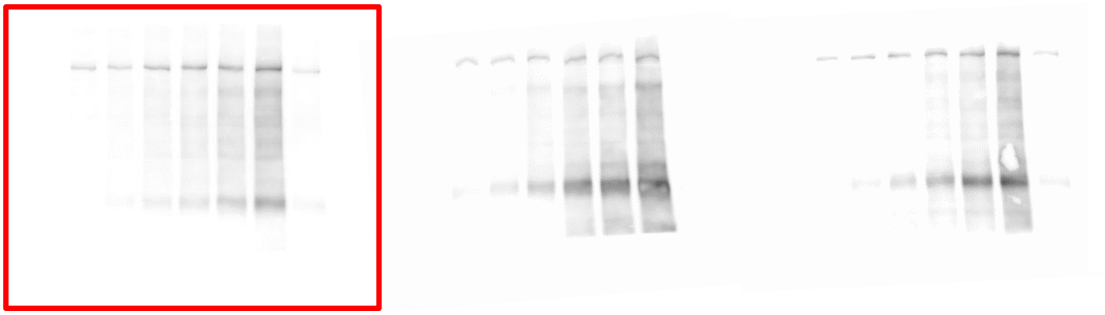

WT TAC 2W

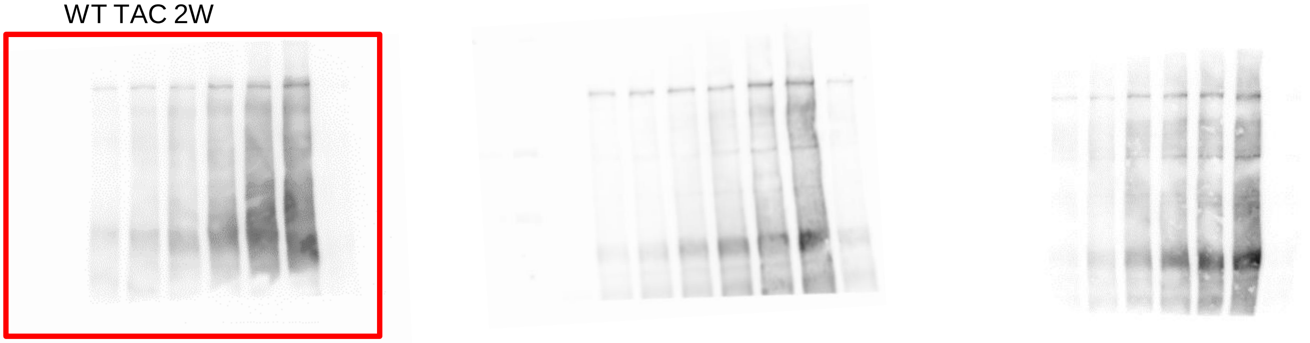

V3599K TAC 2W

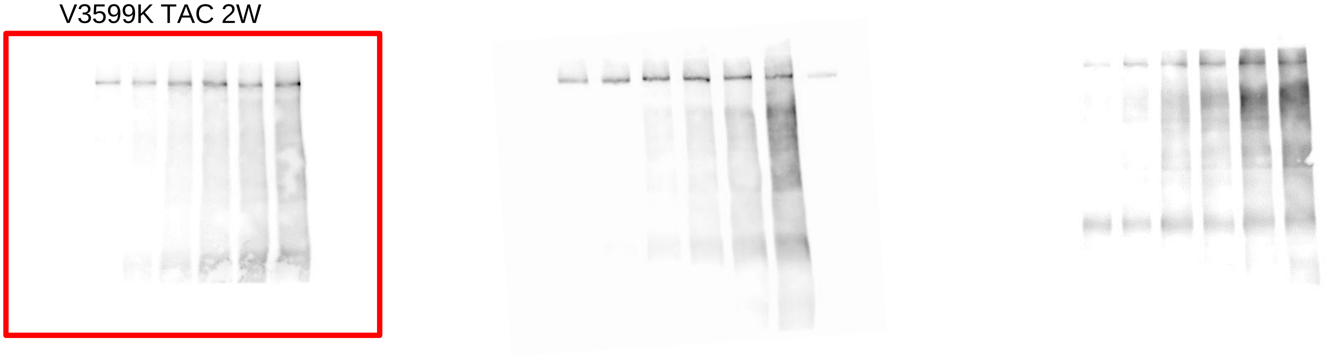

Uncropped gel

The membranes surrounded in red were used for Figure 4e as representative examples.
