# Supplementary material for: Enhancing calmodulin binding to cardiac ryanodine receptor completely inhibits pressure-overload induced hypertrophic signaling
Source: Commun Biol. 2020 Nov 26;3:714. doi: 10.1038/s42003-020-01443-w (PMC7691336; doi:10.1038/s42003-020-01443-w)
Supplement: Supplementary file 2 — Description of Additional Supplementary Files [file 42003_2020_1443_MOESM2_ESM.pdf]

Description of Additional Supplementary Files

File Name: Supplementary Data 1
Description: Data source of Figure 1A-E, 2B, 3A, 3D, 4B-E, 6B-D, 7A, 7B, 8B-E

File Name: Supplementary Data 2
Description: Data source of Figure 5A-C and Supplementary Figure 3, 4A, 4B, 6A.
