# Supplementary material for: Enhancing calmodulin binding to cardiac ryanodine receptor completely inhibits pressure-overload induced hypertrophic signaling
Source: Commun Biol. 2020 Nov 26;3:714. doi: 10.1038/s42003-020-01443-w (PMC7691336; doi:10.1038/s42003-020-01443-w)
Supplement: Supplementary file 5 — Reporting Summary [file 42003_2020_1443_MOESM5_ESM.pdf]

nature research

Corresponding author(s): Masafumi Yano

Last updated by author(s): 2020 - 10 - 08

Reporting Summary

Nature Research wishes to improve the reproducibility of the work that we publish. This form provides structure for consistency and transparency in reporting. For further information on Nature Research policies, see Authors & Referees and the Editorial Policy Checklist .

Statistics

For all statistical analyses, confirm that the following items are present in the figure legend, table legend, main text, or Methods section.

n/a	Confirmed	
☐	☒	The exact sample size (*n*) for each experimental group/condition, given as a discrete number and unit of measurement
☐	☒	A statement on whether measurements were taken from distinct samples or whether the same sample was measured repeatedly
☐	☒	The statistical test(s) used AND whether they are one- or two-sided<br>*Only common tests should be described solely by name; describe more complex techniques in the Methods section.*
☒	☐	A description of all covariates tested
☒	☐	A description of any assumptions or corrections, such as tests of normality and adjustment for multiple comparisons
☐	☒	A full description of the statistical parameters including central tendency (e.g. means) or other basic estimates (e.g. regression coefficient) AND variation (e.g. standard deviation) or associated estimates of uncertainty (e.g. confidence intervals)
☐	☒	For null hypothesis testing, the test statistic (e.g. *F*, *t*, *r*) with confidence intervals, effect sizes, degrees of freedom and *P* value noted<br>*Give P values as exact values whenever suitable.*
☒	☐	For Bayesian analysis, information on the choice of priors and Markov chain Monte Carlo settings
☒	☐	For hierarchical and complex designs, identification of the appropriate level for tests and full reporting of outcomes
☒	☐	Estimates of effect sizes (e.g. Cohen's *d*, Pearson's *r*), indicating how they were calculated

Our web collection on statistics for biologists contains articles on many of the points above.

Software and code

Policy information about availability of computer code

Data collection	Ion Optix, MA, USA<br>BZ9000, Keyence, Japan<br>LSM-510, Carl Zeiss<br>Axiovert 100, Carl Zeiss<br>Illumina NEXTseq500
Data analysis	GraphPad Prism 5.00<br>ImageJ2/FIJI<br>SparkMaster (https://sites.google.com/site/sparkmasterhome/)<br>LabChart AD INSTRUMENTS<br>Principal Component Analysis of JMP Pro ver.14.0 software

For manuscripts utilizing custom algorithms or software that are central to the research but not yet described in published literature, software must be made available to editors/reviewers. We strongly encourage code deposition in a community repository (e.g. GitHub). See the Nature Research guidelines for submitting code & software for further information.

Data

Policy information about availability of data

All manuscripts must include a data availability statement. This statement should provide the following information, where applicable:

- Accession codes, unique identifiers, or web links for publicly available datasets
- A list of figures that have associated raw data
- A description of any restrictions on data availability

All data supporting the findings of this study are available within the paper and its supplementary information files. The source data are provided as Supplementary Data 1 and Supplementary Data 2. In addition, RNA-seq data is deposited in Gene expression omnibus (GEO) (GEO accession no.: GSE158536).

Field-specific reporting

Please select the one below that is the best fit for your research. If you are not sure, read the appropriate sections before making your selection.

- [x] Life sciences
- [ ] Behavioural & social sciences
- [ ] Ecological, evolutionary & environmental sciences

For a reference copy of the document with all sections, see nature.com/documents/nr-reporting-summary-flat.pdf

Life sciences study design

All studies must disclose on these points even when the disclosure is negative.

Sample size	Summarized data of cell width, cell length, and cell area in isolated cardiomyocytes. N= 250-400 cells from 3-5 hearts.Summarized data of sarcomere shortening, peak Ca2+ transient, time from peak to 70% decline of Ca2+ transient, and sarcomere shortening. N= 22-31 cells from 3-6 hearts. Summarized data of spontaneous Ca2+ spark frequency. N= 20-40 cells from 3-5 hearts.Summarized data of SR Ca2+ content measured from caffeine-induced Ca2+ transient. N= 12-15 cells from 3-5 hearts.The immuno-fluorescence signal of the Z-line bound CaM.N= 20-38 cells from 3-4 hearts.Translocations of HDAC. N= 42-60 cells from 3 hearts.Translocations of NFAT. N= 47-58 cells from 3 hearts<br>The experiments described in this exploratory study were done for the first time.<br>No pre-specified effect size could be determined in advance. A plurality of cells were used per mouse, and the average value was used, and 3-5 mice were used to obtain a statistically significant difference.
Data exclusions	No data were excluded from the study.
Replication	The experimental findings were reliably reproducible as is shown in the paper and described above.
Randomization	Mice were assigned to each experiment randomly, and the order of the group in each experiment was decided randomly.
Blinding	The measurement of LV diameter was performed by echocardiography with blinding method. Other experiments were not blinded.

Reporting for specific materials, systems and methods

We require information from authors about some types of materials, experimental systems and methods used in many studies. Here, indicate whether each material, system or method listed is relevant to your study. If you are not sure if a list item applies to your research, read the appropriate section before selecting a response.

Materials & experimental systems

n/a	Involved in the study	
☐	☒	Antibodies
☒	☐	Eukaryotic cell lines
☒	☐	Palaeontology
☐	☒	Animals and other organisms
☒	☐	Human research participants
☒	☐	Clinical data

Methods

n/a	Involved in the study	
☒	☐	ChIP-seq
☒	☐	Flow cytometry
☒	☐	MRI-based neuroimaging

Antibodies

Antibodies used	anti-CaM antibody (EP799Y, Abcam, 1:250, lot# GR3255110-4)<br>anti-RyR antibody (C3-33, Sigma-Aldrich, 1:500, lot# SLBW 8175)<br>HDAC5 (NBP2-22152, Novus Biologicals, 1:500)<br>NFATc4 (ab3447, Abcam, 1:500, lot# GR3251901-3)<br>Alexa Fluor 488–conjugated goat anti-rabbit (Thermo Fisher Scientific, A11008, lot# 1885240)<br>Alexa Fluor 633–conjugated goat anti-mouse (Thermo Fisher Scientific, A21052, lot# 1845042)

Validation	These antibodies were validated in our previous work. (Oda T, et al. J Mol Cell Crdiol. 2018; 125:87-97. Nakamura Y, et al.JCI Insight. 2019 Jun 6;4(11). pii: 126112)

Animals and other organisms

Policy information about studies involving animals; ARRIVE guidelines recommended for reporting animal research

Laboratory animals	C57BL/6 mice, 10–12 weeks old (male or female), were used in this study. WT C57BL/6 mice were obtained from Japan SLC Inc. V3599K-KI mice were from UNITECH Co. Ltd.
Wild animals	No wild animals were used in this study.
Field-collected samples	No field-collected samples were used in this study.
Ethics oversight	This study conforms to the Guide for the Care and Use of Laboratory Animals published by the US National Institutes of Health (NIH Publication No. 85-23, revised 1996). All animal protocols were approved by the Yamaguchi University School of Medicine Animal Experiment Committee (institutional permission # 23-058).

Note that full information on the approval of the study protocol must also be provided in the manuscript.
